# Profile of Exosomal and Intracellular microRNA in Gamma-Herpesvirus-Infected Lymphoma Cell Lines

**DOI:** 10.1371/journal.pone.0162574

**Published:** 2016-09-09

**Authors:** Shiho Hoshina, Tsuyoshi Sekizuka, Michiyo Kataoka, Hideki Hasegawa, Hiromichi Hamada, Makoto Kuroda, Harutaka Katano

**Affiliations:** 1 Department of Pathology, National Institute of Infectious Diseases, 1-23-1 Toyama, Shinjuku-ku, Tokyo 162-8640, Japan; 2 Pathogen Genomic Center, National Institute of Infectious Diseases, 1-23-1 Toyama, Shinjuku-ku, Tokyo 162-8640, Japan; 3 Department of Pediatrics, Yachiyo Medical Center, Tokyo Women’s Medical University, 477-96 Owada-Shinden, Yachiyo, Chiba 276-0046, Japan; Keck School of Medicine of the University of Southern California, UNITED STATES

## Abstract

Exosomes are small vesicles released from cells, into which microRNAs (miRNA) are specifically sorted and accumulated. Two gamma-herpesviruses, Kaposi sarcoma-associated herpesvirus (KSHV) and Epstein—Barr virus (EBV), encode miRNAs in their genomes and express virus-encoded miRNAs in cells and exosomes. However, there is little information about the detailed distribution of virus-encoded miRNAs in cells and exosomes. In this study, we thus identified virus- and host-encoded miRNAs in exosomes released from KSHV- or EBV-infected lymphoma cell lines and compared them with intracellular miRNAs using a next-generation sequencer. Sequencing analysis demonstrated that 48% of the annotated miRNAs in the exosomes from KSHV-infected cells originated from KSHV. Human mir-10b-5p and mir-143-3p were much more highly concentrated in exosomes than in cells. Exosomes contained more nonexact mature miRNAs that did not exactly match those in miRBase than cells. Among the KSHV-encoded miRNAs, miRK12-3-5p was the most abundant exact mature miRNA in both cells and exosomes that exactly matched those in miRBase. Recently identified EXOmotifs, nucleotide motifs that control the loading of miRNAs into exosomes were frequently found within the sequences of KSHV-encoded miRNAs, and the presence of the EXOmotif CCCT or CCCG was associated with the localization of miRNA in exosomes in KSHV-infected cells. These observations suggest that specific virus-encoded miRNAs are sorted by EXOmotifs and accumulate in exosomes in virus-infected cells.

## Introduction

Exosomes are small membrane vesicles of 50–130 nm in diameter that are released by many cultured cells [1]. They contain not only cellular proteins but also mRNA and small RNA [2]. Small RNA including microRNA (miRNA) is particularly abundant in exosomes. Small RNA in exosomes has been thought to have various cellular functions, suggesting that exosomes are a tool for delivering miRNA to distant cells.

The genomes of large DNA viruses such as herpesviruses include sequences that encode miRNAs [3]. The genomes of two human gamma herpesviruses, Kaposi sarcoma-associated herpesvirus (KSHV, or human herpesvirus 8, HHV-8) and Epstein—Barr virus (EBV), encode at least 12 and 25 pri-miRNAs, respectively [4, 5]. Virus-encoded miRNAs have been demonstrated to have various functions and to be associated with oncogenesis and viral replication [3, 6–8]. It has also been demonstrated that viral small RNA including miRNA is present along with host-encoded miRNA in exosomes released from virus-infected cells [9–11]. However, there is no information about the distributions of miRNA in cells and exosomes in virus-infected cells. Moreover, the detailed functions of the virus-encoded miRNA found in exosomes are unknown [11].

Recently, it was demonstrated that some miRNAs are selectively sorted into exosomes, and sequence motifs (EXOmotifs) that control their localization into exosomes were identified within miRNAs [12]. The human hnRNPA2B1 protein plays an important role in miRNA sorting into exosomes by binding specifically to the EXOmotifs in miRNAs. In this study, we revealed the distribution of virus- and host-encoded miRNAs between the exosomes and cellular fraction in KSHV- or EBV-infected lymphoma cell lines using a next-generation sequencer. The miRNA profile revealed that virus-encoded miRNAs were expressed at high levels in exosomes. In addition, we identified EXOmotifs that effectively promoted the loading of miRNAs into exosomes within virus- and host-encoded miRNAs in the virus-infected cells.

## Materials and Methods

### Cell lines

Two KSHV-infected lymphoma cell lines, BCBL-1 and TY-1, an EBV-infected lymphoblastoid cell line (LCL), and Bjab, an EBV- and KSHV-negative Burkitt lymphoma cell line, were cultured in 200 mL of RPMI 1640 with 5% fetal bovine serum (depleted of bovine exosomes by overnight centrifugation at 100,000×g), 100 U/ml penicillin, and 100 μg/ml streptomycin [13, 14].

### Exosome isolation

Exosomes were isolated following a previously described ultracentrifugation protocol [15]. After incubation for 72 h at 37°C with 5% CO_2_, cells were centrifuged at 300×g for 5 min. The supernatant was then passed through a 0.22-μm filter. This filtered supernatant was transferred to a fresh tube (50 mL) and centrifuged at 2,000×g for 30 min. The supernatant obtained from this procedure was then transferred to ultracentrifuge tubes and spun in a SW32Ti swinging bucket rotor (Beckman Coulter, Brea, CA, USA) at 12,000×g for 30 min at 4°C. The supernatant was again transferred to new ultracentrifuge tubes and spun for 70 min at 110,000×g. The supernatant was then discarded and the pellet was suspended in 1 ml of sterile phosphate-buffered saline (PBS). Samples were then transferred to 1.5-ml microtubes and supplemented with 200 μL of ExoQuick-TC (System Biosciences, Mountain View, CA, USA). After incubation at 4°C overnight, the mixture was centrifuged at 1,500×g for 30 min. The supernatant was then discarded and the pellet was centrifuged at 1,500×g for 5 min. Finally, the resulting pellet was suspended in 100 μL of sterile PBS.

### Electron microscopy

A 6-μL aliquot of exosomes was absorbed onto glow-discharged 300-mesh heavy-duty carbon-coated Cu grids (Veco grids; Nisshin EM, Tokyo, Japan) for 2 min and the excess was blotted on filter paper (Whatman; GE Healthcare, Piscataway, NJ, USA). The grids were then washed twice with MilliQ water and negatively stained with 2% uranyl acetate. Data were collected using an H7700 transmission electron microscope (Hitachi, Tokyo, Japan) operating at 80 kV and 10,000× magnification.

### PCR

Virus particles of KSHV and EBV were collected from filtered supernatants of 12-O-Tetradecanoylphorbol 13-acetate-stimulated BCBL-1 and B95-8 cells by ultracentrifugation [16]. A 10-μL aliquot of exosomes and virus particles were treated with DNase (Turbo DNase, Ambion, Austin, TX, USA), in accordance with the manufacturer’s protocol. DNase was inactivated by heat-incubation at 70°C for 5 min. DNA was extracted from DNase-treated exosomes/virus particles and Bjab cell using the DNeasy Blood & Tissue Kit (Qiagen, Hilden, Germany). DNA fragments of KSHV (KS330_233_), EBV (W-region), and human beta-globin were amplified by PCR as described previously [17–19].

### Western blotting analysis

A 10-μL aliquot of exosomes/ virus or 1× 10^5^ cells was lysed with 2× sodium dodecyl sulfate sample buffer, applied to a well of sodium dodecyl sulfate-polyacrylamide gel electrophoresis, and transferred onto a polyvinylidene fluoride microporous membrane (Immobilon-P Transfer Membrane, Merck Millipore, Bedford, MA, USA) using the NuPAGE system (Life Technologies, Carlsbad, CA, USA). The membranes were blocked with Block Ace and probed with primary antibodies, anti-KSHV ORF45 (2D4A5, Abcam plc, Cambridge, UK), anti-CD63 (H-193, Santa Cruz Biotechnology, Santa Cruz, CA, USA), anti-HSP70 (System Biosciences, Palo Alto, CA, USA), or anti-Lyn (sc-7274, Santa Cruz Biotechnology) as markers of exosome [20–22]. After washing, the membranes were incubated with horse radish peroxidase-conjugated anti-mouse or anti-rabbit antibodies (Promega, Madison, WI, USA) with an immunoreaction enhancer solution (Can Get Signal, Toyobo, Osaka, Japan). Blots were visualized by Super-Signal West Femto Chemiluminescent Substrate (Thermo Fisher Scientific, Waltham, MA, USA) and images were captured with a C-Digit Blot Scanner (LI-COR biosciences, Lincoln, NE, USA).

### RNA extraction

Total RNA was extracted from cells or exosomes using the High Pure miRNA Isolation Kit (Roche Diagnostics, Boehringer Mannheim, Germany), in accordance with the manufacturer’s protocol. The extracted RNA was analyzed using small RNA chips on a 2100 Bioanalyzer (Agilent Technologies, Santa Clara, CA, USA). Small RNA profiles contained small RNA of between 4 and 40 nucleotides in length, which is consistent with miRNA.

### Next-generation sequencing (NGS)

The TruSeq Small RNA-Seq Sample Prep Kit (Illumina, San Diego, CA, USA) was used in accordance with the manufacturer’s protocol to prepare a small RNA library for sequencing. The quality and yield after sample preparation were measured using Bioanalyzer with a High Sensitivity DNA kit (Agilent Technologies) and corresponded to the expected 150 bp. DNA sequencing was carried out using Miseq (Illumina, San Diego, CA, USA) with MiSeq reagent kit v3, in accordance with the manufacturer’s protocol. Sequence reads were analyzed with CLC Genomics Workbench (version 9.0; Qiagen). After adaptor trimming, reads of less than 15 or more than 26 nucleotides in length were removed, and all reads of 15–25 nucleotides in length were analyzed against miRBase release 21 retrieved from the miRNA database (http://www.mirbase.org/). Homo_sapiens.GRCh37.57.ncrna was used as a comprehensive noncoding RNA database (http://www.ncrna.org/). All annotated reads matching pre-miRNA were counted as miRNA reads.

### Real-time RT-PCR for miRNA

Copy numbers of human miRNAs were measured by ABI TaqMan^®^ MicroRNA Assays in accordance with the manufacturer’s protocol (Applied Biosystems, Foster City, CA, USA). In addition, the quantification of 17 KSHV-encoded miRNAs and a human cellular miRNA, miR21, was performed using the miScript Reverse Transcription Kit with miScript Primer Assays and miScript SYBR Green PCR Kit from Qiagen.

### Statistical analysis

Data were analyzed by Student’s t-test, Mann-Whitney U-test, or Chi-square test using SPSS software (IBM, Armonk, NY, USA).

### Accession number

Sequence data of the small RNAs analyzed by NGS in this study were deposited in the DNA Data Bank of Japan (DDBJ; accession number: DRA004793; Bioproject PRJDB4918).

## Results

### Profile of miRNAs by NGS

**Fig 1:**
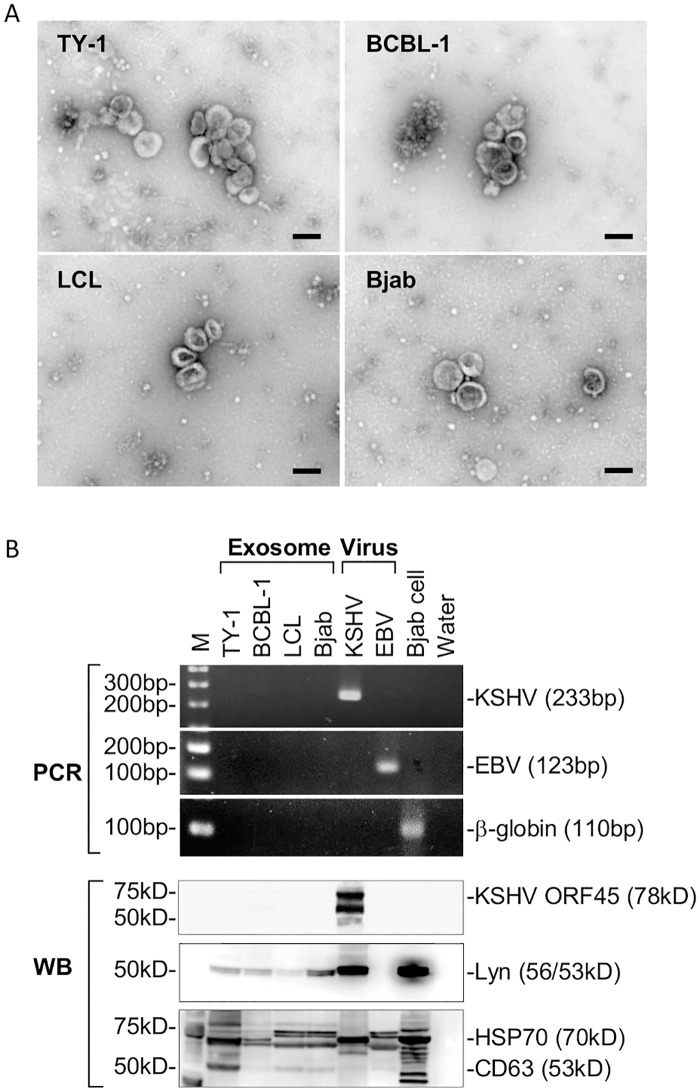
Isolation of exosomes. (A) Representative transmission electron microscopy images of exosome samples. Exosomes were isolated by ultracentrifugation and ExoQuick, and analyzed by electron microscopy. Scale bar = 100 nm. (B) PCR and western blotting analysis of isolated exosomes. Virus particles of KSHV and EBV and cell lysate of Bjab cells were also examined. KSHV KS330_233_, EBV W and beta-globin genes were amplified with PCR (upper panels). A faint band of KSHV is found in the exosome sample from BCBL-1 in PCR. KSHV ORF45, Lyn, CD63, HSP70, and Lyn were detected in isolated exosome samples with western blotting analysis (lower panels). A faint band of KSHV ORF45 is observed in the exosome sample from BCBL-1.

To reveal the miRNA profiles in exosomes and cells, small RNAs were extracted from exosomes and cells separately. Exosomes were derived from the supernatant of TY-1, BCBL-1, LCL, and Bjab cell cultures using Exo-Quick; transmission electron microscopy showed the presence of exosomes in the final pellet of Exo-Quick (Fig 1). No viral particle was observed in the exosome samples with electron microscopy. PCR analysis for KSHV and EBV DNA showed no or faint band in the exosome samples, suggesting no or low level of contamination of virus particles in the isolated exosomes (Fig 1). The small RNAs from the exosomes and cells were sequenced using a next-generation sequencer (Table 1). Small RNAs isolated from cells and exosomes were subjected to NGS, yielding averages of 1,712,881 and 1,536,143 reads for cellular and exosomal RNAs, respectively. The sequences that could be aligned to the reference genome represented 29.76–60.28% of exosomal miRNA reads and 54.81–88.42% of cellular miRNA reads (Table 1 and S1 Table). In KSHV-infected cell lines, TY-1 and BCBL-1, 48% of the annotated miRNAs in the exosomes originated from KSHV, while the proportion was 43–44% in cells. In EBV-transformed LCL, 7% of annotated miRNAs in the exosomes originated from EBV, while the proportion was 15.7% in cells.

**Table 1:** Read numbers of miRNA in exosome and cell by NGS.

		Exosome				Cell			
		TY1	BCBL1	LCL	Bjab	TY1	BCBL1	LCL	Bjab
**Annotated**		1,132,921	988,599	939,067	193,012	1,029,586	1,668,043	1,193,764	475,273
**with miRBase**		514,176	374,262	292,727	31,110	489,017	939,895	1,146,054	242,777
	**Homo sapiens**	263,963	194,190	271,180	30,988	278,734	521,351	965,346	242,667
	**KSHV**	250,123	180,058	624	120	210,281	418,538	3	108
	**EBV**	90	14	20,923	2	2	6	180,705	2
**with Homo_sapiens.GRCh37.57.ncrna**		618,745	614,337	646,340	161,902	540,569	728,148	47,710	232,496
**Unannotated**		1,237,378	1,286,017	618,882	455,651	326,463	903,205	156,328	391,913
**Total**		2,370,299	2,274,616	1,557,949	648,663	1,356,049	2,571,248	1,350,092	867,186
**Annotated (%)**		47.80%	43.46%	60.28%	29.76%	75.93%	64.87%	88.42%	54.81%
**with miRBase(%)**		45.38%	37.86%	31.17%	16.12%	47.50%	56.35%	96.00%	51.08%
	**Homo sapiens**	51.34%	51.89%	92.64%	99.61%	57.00%	55.47%	84.23%	99.95%
	**KSHV**	48.65%	48.11%	0.21%	0.39%	43.00%	44.53%	0.00%	0.04%
	**EBV**	0.02%	0.00%	7.15%	0.01%	0.00%	0.00%	15.77%	0.00%
**with Homo_sapiens.GRCh37.57.ncrna**		54.62%	62.14%	68.83%	83.88%	52.50%	43.65%	4.00%	48.92%
**Unannotated**		52.20%	56.54%	39.72%	70.24%	24.07%	35.13%	11.58%	45.19%
**Total**		100.00%	100.00%	100.00%	100.00%	100.00%	100.00%	100.00%	100.00%

### Distribution of miRNAs in cells and exosomes

**Fig 2:**
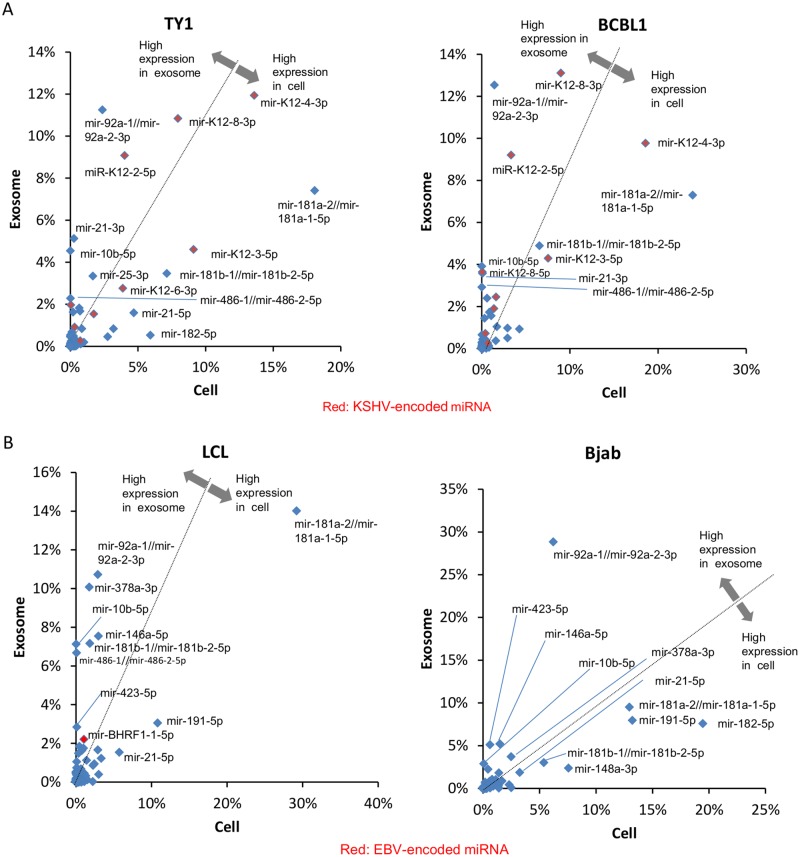
miRNA expression between cells and exosomes by NGS. Horizontal and vertical axes indicate the proportions of miRNAs in cells and exosomes, respectively. Black line in the center of each graph represents a 50:50 split between cells and exosomes. Red dots represent virus-encoded miRNAs. (A) TY-1 and BCBL-1, (B) LCL and Bjab.

**Fig 3:**
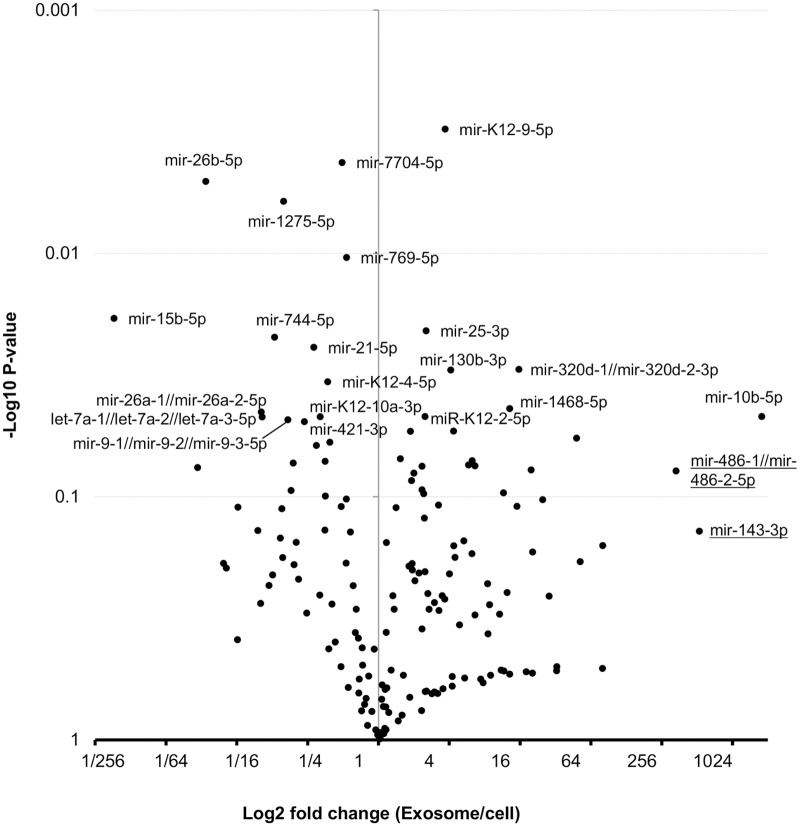
Volcano plot of exosomal and intracellular miRNA expression in KSHV-infected cells. Fold changes of miRNA expression between exosomes and cells of TY-1 and BCBL-1; P values are shown. miRNAs with a significant difference at P<0.05 are labeled in black. miRNAs with more than 200-fold difference between exosomes and cells are underlined.

**Fig 4:**
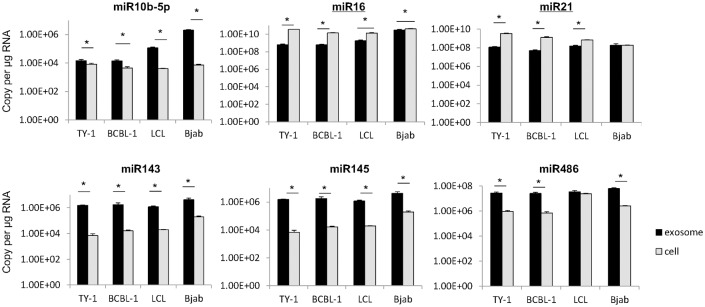
miRNA expression in exosomes and cells was confirmed by real-time RT-PCR. Six miRNAs were examined by ABI TaqMan^®^ MicroRNA Assays. miR10b-5p, miR143, miR145, and miR486 were highly expressed in exosomes, but the levels of miR16 and miR21 were higher in the cellular component. Asterisk indicates a significant difference.

All mature annotated miRNAs were ranked according to their read counts in cells and exosomes of TY-1 and BCBL-1 (Tables 2 and 3). The most abundant mature miRNAs in exosomes were miRK12-4-3p in TY-1 and miRK12-8-3p in BCBL-1. In both of these cell lines, KSHV miRNAs were the most abundant mature miRNA. Eight and seven KSHV-encoded miRNAs ranked in the top 20 in exosomes of TY-1 and BCBL1, respectively. These KSHV-encoded miRNAs also had high rankings among the cellular miRNAs. Identified miRNAs were plotted based on the percentages of total read counts (Fig 2). miR-K12-4-3p, miR-K12-8-3p, miR-K12-2-5p, and miR-K12-3-5p were frequently expressed in both exosomes and cells of TY-1 and BCBL-1 (Fig 2A). miR-92a ranked second for exosomes but twelfth and thirteenth for cells in TY-1 and BCBL-1, respectively. Higher expression of miR-92a was also observed in LCL and Bjab cells (S2 and S3 Tables). miRNAs with different levels of expression between exosomes and cells are listed in S4 and S5 Tables. A volcano plot shows that 22 miRNAs were identified as exhibiting significantly different expression between cells and exosomes (P<0.05, Fig 3). The levels of miR143-3p, miR-486-5p, and miR-10b-5p were more than 200 times higher in exosomes than in cells (Fig 3). The high expression levels of these host-encoded miRNAs in exosomes were confirmed by real-time PCR (ABI TaqMan^®^ MicroRNA Assays) in KSHV-infected cells (Fig 4). In addition, higher expression of miR16 and miR21 in cells than in exosomes was demonstrated by real-time PCR, except for in Bjab cells. In EBV-infected LCL, high expression of miR10b-5p, miR143, and miR145 was confirmed in exosomes by real-time PCR (Fig 4).

**Table 2:** Top 20 miRNAs in exosome of TY-1. Ranks are indicated for exosomes and cells. Exomotif and cell motif are indicated by underline and italic, respectively. KSHV-encoded miRNAs are indicated by bold.

ID	Mature sequence	Rank in exosome	Rank in cell
**mir-K12-4-3p**	TAGAATACTGAGGCCTAGCTGA	1	2
mir-92a-1//mir-92a-2-3p	TATTGCACTTGTCCCGGCCTGT	2	12
**mir-K12-8-3p**	CTAGGCGCGACTGAGAGAGCA	3	4
**miR-K12-2-5p**	AACTGTAGTCCGGGTCGATCTG	4	8
mir-181a-2//mir-181a-1-5p	*AACATT*CAACGCTGTCGGTGAGT	5	1
mir-21-3p	CAACACCAGTCGATGGGCTGT	6	28
**mir-K12-3-5p**	TCACATTCTGAGGACGGCAGCGA	7	3
mir-10b-5p	TACCCTGTAGAACCGAATTTGTG	8	159
mir-181b-1//mir-181b-2-5p	*AACATT*CATTGCTGTCGGTGGGT	9	5
mir-25-3p	CATTGCACTTGTCTCGGTCTGA	10	14
**mir-K12-6-3p**	TGATGGTTTTCGGGCTGTTGAG	11	9
mir-486-1//mir-486-2-5p	TCCTGTACTGAGCTGCCCCGAG	12	126
**mir-K12-8-5p**	ACTCCCTCACTAACGCCCCGCT	13	77
mir-378a-3p	ACTGGACTTGGAGTCAGAAGGC	14	20
mir-92b-3p	TATTGCACTCGTCCCGGCCTCC	15	19
mir-320a-3p	AAAAGCTGGGTTGAGAGGGCGA	16	36
mir-21-5p	TAGCTTATCAGACTGATGTTGA	17	7
**mir-K12-5-3p**	TAGGATGCCTGGAACTTGCCGGT	18	13
**mir-K12-7-3p**	TGATCCCATGTTGCTGGCGC	19	25
mir-30d-5p	TGTA*AACATC*CCCGACTGGAAG	20	16

**Table 3:** Top 20 miRNAs in exosome of BCBL-1. Ranks are indicated for exosomes and cells. Exomotif and cell motif are indicated by underline and italic, respectively. KSHV-encoded miRNAs are indicated by bold.

ID	Mature sequence	Rank in exosome	Rank in cell
**mir-K12-8-3p**	CTAGGCGCGACTGAGAGAGCA	1	3
mir-92a-1//mir-92a-2-3p	TATTGCACTTGTCCCGGCCTGT	2	13
**mir-K12-4-3p**	TAGAATACTGAGGCCTAGCTGA	3	2
**miR-K12-2-5p**	AACTGTAGTCCGGGTCGATCTG	4	7
mir-181a-2//mir-181a-1-5p	*AACATT*CAACGCTGTCGGTGAGT	5	1
mir-181b-1//mir-181b-2-5p	*AACATT*CATTGCTGTCGGTGGGT	6	5
**mir-K12-3-5p**	TCACATTCTGAGGACGGCAGCGA	7	4
mir-10b-5p	TACCCTGTAGAACCGAATTTGTG	8	185
**mir-K12-8-5p**	ACTCCCTCACTAACGCCCCGCT	9	55
mir-21-3p	CAACACCAGTCGATGGGCTGT	10	50
mir-486-1//mir-486-2-5p	TCCTGTACTGAGCTGCCCCGAG	11	117
**mir-K12-6-3p**	TGATGGTTTTCGGGCTGTTGAG	12	11
mir-25-3p	CATTGCACTTGTCTCGGTCTGA	13	20
**mir-K12-5-3p**	TAGGATGCCTGGAACTTGCCGGT	14	14
mir-378a-3p	ACTGGACTTGGAGTCAGAAGGC	15	16
mir-92b-3p	TATTGCACTCGTCCCGGCCTCC	16	15
mir-320a-3p	AAAAGCTGGGTTGAGAGGGCGA	17	25
mir-30d-5p	TGTA*AACATC*CCCGACTGGAAG	18	10
mir-191-5p	CAACGGAATCCCAAAAGCAGCTG	19	9
mir-21-5p	TAGCTTATCAGACTGATGTTGA	20	6

### Mature miRNAs in exosomes and cells exactly or not exactly matching sequences registered in miRBase

**Fig 5:**
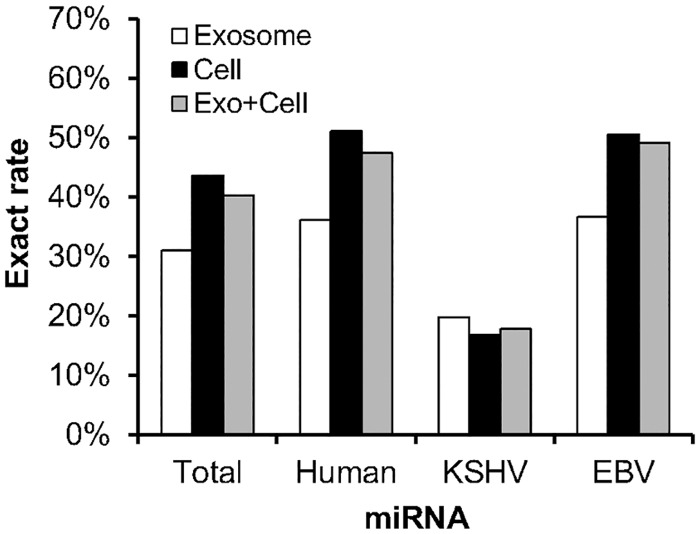
Expression of exact and nonexact mature miRNAs among host-, KSHV-, and EBV-encoded miRNAs by NGS. The exact rate was calculated by dividing each exact miRNA read number by the total exact and nonexact miRNA read number of four cell lines, TY-1, BCBL-1, LCL, and Bjab. Read numbers of exact mature miRNA in each cell are shown in Table 4.

**Fig 6:**
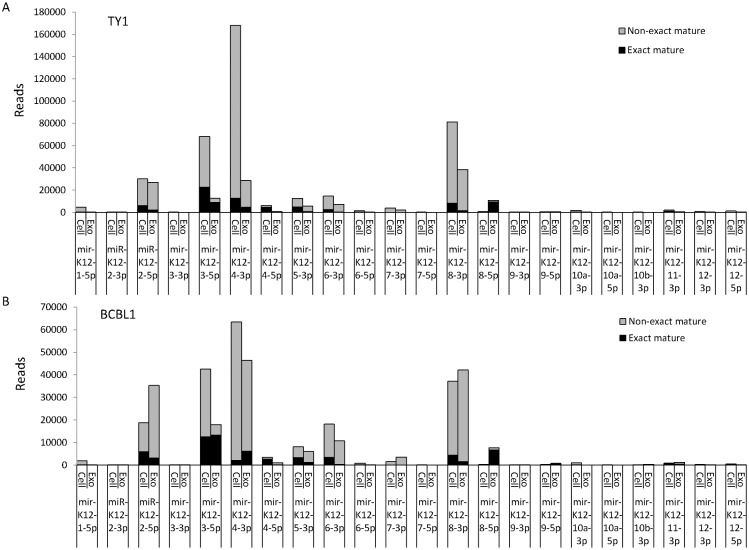
Expression of exact and nonexact mature miRNAs in KSHV-infected cells by NGS. Reads of exact and nonexact mature miRNAs were counted in TY-1 (A) and BCBL-1 (B) cells. miRK12-3-5P was the most abundant KSHV exact mature miRNA in both cells and exosomes.

**Fig 7:**
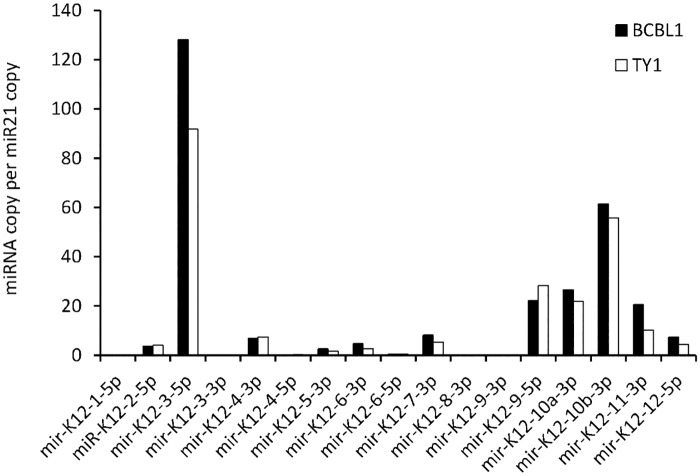
Expression of mature miRNAs in KSHV-infected cells by real-time PCR. High expression of exact mature miRK12-3-5p in exosomes was confirmed by real-time PCR (miScript PCR assay, Qiagen).

**Fig 8:**
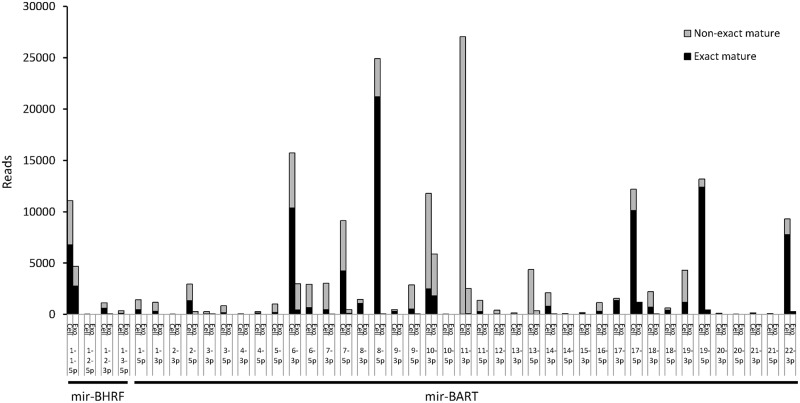
Expression of exact and nonexact mature miRNAs in LCL by NGS. miR-BART8-5p was the most abundant EBV exact mature miRNA in cells, but it was expressed at a low level in exosomes of LCL.

The mature miRNAs included both exact mature miRNAs, which corresponded exactly to sequences registered in miRBase, and nonexact mature miRNAs, which had a deletion, addition, and/or mutation causing them to differ from the exact sequences. NGS revealed that exosomes contained nonexact mature miRNAs more frequently than cells (Table 4). The proportion of exact mature miRNAs was 27–49% in exosomes, which was significantly lower than that in cells (34–56%, P<0.001, Chi-square test). KSHV miRNAs included nonexact mature miRNAs at a higher rate than EBV- and host-encoded miRNAs (Fig 5). Exact mature miRNAs were more common in cells than in exosomes for EBV- and host-encoded miRNAs, but not for KSHV-encoded miRNAs (Fig 5). High total read numbers of miRK12-4-3p and miRK12-8-3p were detected in TY1 and BCBL1, but they contained exact mature miRNAs at a low rate (Fig 6). NGS data indicated that the most abundant exact miRNA in KSHV-encoded miRNA was miRK12-3-5p in TY1 and BCBL1 (Fig 6). High expression of exact mature miRK12-3-5p in the exosomes was confirmed by miScript PCR assay real-time PCR, which can efficiently detect exact mature miRNAs (Fig 7). A similar phenomenon was observed for EBV-encoded miRNAs. NGS revealed that these miRNAs included nonexact mature miRNAs at 38%–50% (Fig 5). The proportions of exact and nonexact mature miRNAs varied among the miRNAs (Fig 8). In addition, some EBV-encoded miRNAs differed in their expression levels between cells and exosomes (Fig 8). For example, miR-BART8-5p was found to be the most abundant EBV exact mature miRNA in cells, but was expressed at a low level in exosomes of LCL.

**Table 4:** Read numbers of exact mature miRNAs in exosome and cell. Exosome miRNA contained non-exact mature miRNAs more frequently than intracellular miRNAs.

Cell	Exosome			Cell		
	Exact	Mature total	Exact rate	Exact	Mature total	Exact rate
TY1	107,769	389,307	28%	157,241	467,456	34%
BCBL1	78,287	293,304	27%	310,412	905,947	34%
LCL	102,571	267,562	38%	590,120	1,120,953	53%
Bjab	14,373	29,071	49%	133,102	238,784	56%

### EXOmotifs and miRNA localization

**Fig 9:**
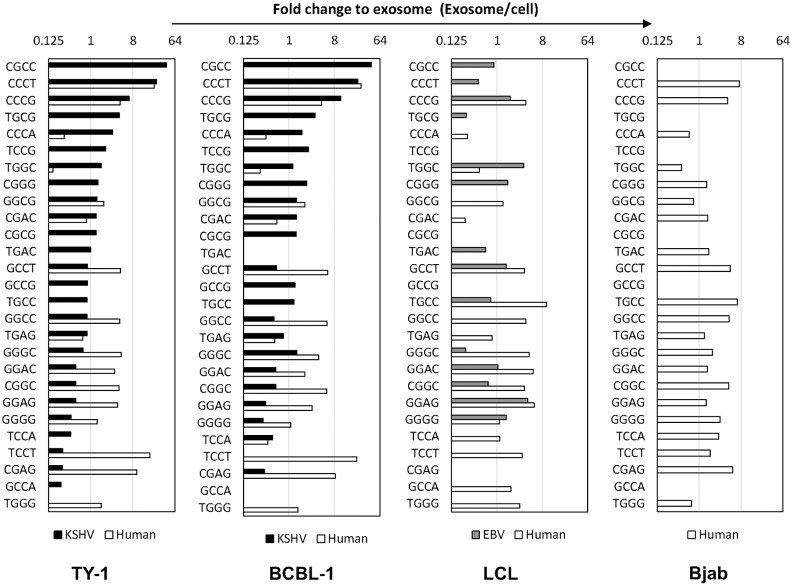
Fold changes between exosomes and cells of miRNAs containing EXOmotifs. Fold changes of miRNAs containing EXOmotifs for host-, KSHV-, and EBV-encoded miRNAs are shown. The fold change for each EXOmotif was calculated by dividing the read number in exosomes by that in cells of miRNAs with the EXOmotif.

A recent study identified several short sequence motifs (EXOmotifs) within miRNAs that guide their loading into exosomes [12]. NGS revealed that EXOmotifs were common within the sequences of the top 20 mature miRNAs found in exosomes of TY-1 and BCBL-1 (Tables 2 and 3). Next, to identify EXOmotifs within these miRNAs that efficiently promote sequestration into exosomes, we counted the reads of exosomal and intracellular mature miRNAs with specific EXOmotifs. Then, we calculated the fold change of miRNA expression between exosomes and cells for each EXOmotif by dividing the read number in exosomes by the read number in cells of miRNAs with the EXOmotif. The fold change between exosomes and cells varied among EXOmotifs (Fig 9). Two EXOmotifs, “CCCG” and “CCCT,” were associated with high fold change to exosomes of both KSHV- and host-encoded miRNAs. This supports the findings of a previous study in which these two motifs were characterized as EXOmotifs that strongly promote the transit of miRNAs into exosomes [12]. Several other EXOmotifs showed more than one fold change for host-encoded miRNAs, but not for KSHV-encoded miRNAs. Interestingly, two motifs (CGCC and TGCG) observed within KSHV- and EBV-encoded miRNAs, but not within host-encoded miRNAs, showed more than four fold change to exosomes in KSHV-infected cells.

## Discussion

In this study, we demonstrated the distribution of virus- and host-encoded miRNAs between the exosomes and the cellular fraction in KSHV- or EBV-infected lymphoma cell lines using a next-generation sequencer. Approximately half of the annotated miRNAs in the exosomes of KSHV-infected cells originated from KSHV, whereas 7% of the annotated miRNAs in the exosomes of EBV-infected cells originated from EBV. Among the KSHV-encoded miRNAs, miR-K12-8-3p, miR-K12-4-3p, and miR-K12-2-5p were expressed highly in the exosomes of KSHV-infected cells. Moreover, miR-92a, mir-10b-5p, and mir-143-3p were identified as exosomal host-encoded miRNAs as their levels in exosomes were more than double those in cells. Exosomes contained mature miRNAs not exactly matching miRBase more frequently than cells. miR-K12-3-5p was identified as the most common KSHV-encoded exact mature miRNA in exosomes. The EXOmotifs “CCCG” and “CCCT” are likely to be associated with the localization of miRNAs in exosomes in KSHV-infected cells.

Previous studies on serum samples showed that exosomes contained virus-encoded miRNAs (EBV, KSHV, JCV, and HCV) [15, 23–25]. However, the detailed profiles of virus-encoded miRNAs in both exosomes and intracellularly have not been reported yet. This study showed the profile of exosomal and intracellular miRNAs in gamma-herpesvirus-infected lymphoma cell lines. As shown in Table 1, 48% of the annotated miRNAs in the exosomes originated from viruses, but the corresponding proportion was 43–44% in the cells of TY1 and BCBL1. This proportion is markedly higher than that for EBV miRNAs in EBV-infected cells. In EBV-infected LCL, the proportion of EBV miRNAs was only 7.15% in exosomes compared with 15.8% in cells. The higher proportion of KSHV miRNAs in exosomes than in cells suggests that there is a selective mechanism by which KSHV miRNAs are sorted into the exosomes. As shown in Tables 2 and 3, KSHV miRNAs identified abundantly in the exosomes frequently contained an EXOmotif within their sequences. Two of them, CGCC and TGCG, showing more than fourfold change of exosome/cell, were found only within KSHV-encoded miRNAs, but not within host-encoded miRNAs, in the exosomes among the top 20 miRNAs found in this location. In a previous study, it was demonstrated that CCCT and CCCG motifs were under the control of sumoylated hnRNPA2B1 in mammalian cells, for sorting to the exosomes [12]. It is assumed that KSHV-encoded miRNAs use a special sorting mechanism other than that for host-encoded miRNAs. An EXOmotif “CCCT” did not show an enrichment in exosomes of LCL (Fig 9). Moreover, Tables 2 and 3 shows that some miRNAs in exosome have no EXOmotif in their sequences. It has been reported that short nucleotide sequences such as miRNA guides the transport of RNAs to different subcellular compartments by various mechanisms [26–28]. For example, the terminal motif of miR-29b causes the nuclear enrichment of this miRNA [27]. Our observations on virus miRNAs in exosomes suggested the presence of unknown mechanism other than EXOmotif-dependent enrichment of miRNAs in exosomes.

NGS data clearly demonstrated that mature miRNAs expressed in both cells and exosomes contained nonexact mature miRNAs at a high rate. It should be noted that such nonexact forms of miRNA with the addition or deletion of nucleotides at the 3’ end can theoretically not be detected by stem-looped real-time PCR [29]. To date, only exact mature miRNAs have been detected in quantitative studies using stem-looped real-time PCR [30]. Indeed, real-time PCR demonstrated a similar profile of miRNAs to that of exact mature miRNAs by NGS in part (Figs 6 and 7). However, there is a large difference between the two because reads of nonexact mature miRNAs were more abundant than those of exact mature miRNAs for most of the KSHV-encoded miRNAs. NGS also revealed that the exosomes contained nonexact mature miRNAs more frequently than cells. This was observed in all of the cells that we tested, suggesting the presence of a mechanism sorting nonexact mature miRNAs to the exosomes. This also suggests that exosomes might have a function to exclude nonexact mature miRNAs from cells and to concentrate mature and functional miRNAs in cells. On the other hand, nonexact miRNAs in exosomes might be delivered with unexpected functions to target cells. Further studies on sorting mechanisms of exosomes are needed to reveal exact functions and biological roles of miRNAs in exosomes.

## Conclusions

In this study, using NGS, we revealed the profile of virus- and host-encoded miRNAs in the exosomes released from KSHV- or EBV-infected lymphoma cell lines. The exosomes from KSHV-infected cells contained KSHV-encoded miRNAs at a high rate. Exosomes contained nonexact mature miRNAs more frequently than cells. EXOmotifs identified as nucleotide motifs that effectively promoted the loading of miRNAs into exosomes were frequently found within the sequences of KSHV-encoded miRNAs. These findings suggest that specific virus-encoded miRNAs are sorted via EXOmotifs and accumulate in the exosomes of KSHV-infected cells. Exosomes have received considerable attention in recent years as they have been suggested to have potential uses as biomarkers, vaccines, and vehicles for gene therapy [31–34]. Information on the profiles of virus- and host-encoded miRNAs and their differences between exosomes and cells should be useful for establishing these potential applications.

## Supporting Information

S1 Table Read numbers of annotated mature miRNAs in samples by NGS. (XLSX)

S2 Table Top 20 miRNAs in exosomes of LCL. Ranks are indicated for exosomes and cells.
(XLSX)

S3 Table Top 20 miRNAs in exosomes of Bjab. Ranks are indicated for exosomes and cells.
(XLSX)

S4 Table miRNAs more than double as abundant in exosomes than in cells. Each number indicates the proportion of total reads. Red indicates miRNAs found in both TY1 and BCBL1.
(XLSX)

S5 Table miRNAs more than double as abundant in cells than in exosomes. Each number indicates the proportion of total reads. Red indicates miRNAs found in both TY1 and BCBL1.
(XLSX)
